# Pathways of care for HIV infected children in Beira, Mozambique: pre-post intervention study to assess impact of task shifting

**DOI:** 10.1186/s12889-018-5646-8

**Published:** 2018-06-07

**Authors:** Claudia Marotta, Carlo Giaquinto, Francesco Di Gennaro, Kajal D. Chhaganlal, Annalisa Saracino, Jorge Moiane, Guido Maringhini, Damiano Pizzol, Giovanni Putoto, Laura Monno, Alessandra Casuccio, Francesco Vitale, Walter Mazzucco

**Affiliations:** 1 Department of Science for Health Promotion and Mother to Child Care “G. D’Alessandro”, University of Palermo, via del vespro, 133, 90127 Palermo, Italy; 2 Department of Women’s and Children’s Health, University of Padova, Padova, Italy; 3 Clinic of Infectious Diseases, University of Bari, Bari, Italy; 4 Center for Research in Infectious Diseases, Faculty of Health Sciences, Catholic University of Mozambique, Beira, Mozambique; 5 Doctors with Africa, Beira, Mozambique; 6 Operational Research Unit, Doctors with Africa, Beira, Mozambique; 7 Operational Research Unit, Doctors with Africa, Padova, Italy

**Keywords:** Human immunodeficiency virus, HIV exposed infants, HIV infected children, Task-shifting, Pathways of care, Paediatric HIV care and treatment

## Abstract

**Background:**

In 2013, Mozambique implemented task-shifting (TS) from clinical officers to maternal and child nurses to improve care for HIV positive children < 5 years old. A retrospective, pre-post intervention study was designed to evaluate effectiveness of a new pathway of care in a sample of Beira District Local Health Facilities (LHFs), the primary, local, community healthcare services.

**Methods:**

The study was conducted by accessing registries of At Risk Children Clinics (ARCCs) and HIV Health Services. Two time periods, pre- and post-intervention, were compared using a set of endpoints. Variables distribution was explored using descriptive statistics. T-student, Mann Whitney and Chi-square tests were used for comparisons.

**Results:**

Overall, 588 HIV infected children (F = 51.4%) were recruited, 330 belonging to the post intervention period. The mean time from referral to ARCC until initiation of ART decreased from 2.3 (± 4.4) to 1.1 (± 5.0) months after the intervention implementation (*p*-value: 0.000). A significant increase of Isoniazid prophylaxis (O.R.: 2.69; 95%CI: 1.7–4.15) and a decrease of both regular nutritional assessment (O.R. = 0.45; 95%CI: 0.31–0.64) and CD4 count at the beginning of ART (O.R. = 0.46; 95%CI: 0.32–0.65) were documented after the intervention.

**Conclusions:**

Despite several limitations and controversial results on nutrition assessment and CD4 count at the initiation of ART reported after the intervention, it could be assumed that TS alone may play a role in the improvement of the global effectiveness of care for HIV infected children only if integrated into a wider range of public health measures.

## Background

In past years, massive effort has been made globally in order to improve maternal and child mortality with regard to Human Immunodeficiency Virus (HIV) care and treatment in countries documenting highest prevalence [1].

Since 2001, according to the Prevention of Mother to Child Transmission (PMCT) Programs promoted by the World Health Organization (WHO) [2], the proportion of HIV infected pregnant women with access to the antiretroviral drugs has constantly increased, achieving 77% (69–86%) coverage worldwide in 2016 [3]. This improvement resulted in an indirect reduction of the number of HIV infected children (0–14), estimated at 150,000 (110,000–190,000) new infections in 2016, globally [3]. However, in 2016, the paediatric antiretroviral treatment (ART) coverage was still at 49% (42–55%) [3].

Mozambique, an east African country, has documented the world’s 6th highest HIV prevalence, with an estimated 12.3% (10.6–13.9%) of adults aged 15–49 and 13,000 (7000–120,000) children under 15 years old “living with” HIV in 2016 [4]. Although the number of new HIV infections has decreased from 160,000 in 2001 to 83,000 in 2016, there are still 13,000 newly infected children every year [4]. Access to ART significantly increased in the country after 2010 due to the decentralization of HIV care from urban hospitals to the spread of local health facilities (LHFs) [3], the healthcare services nearest to the communities. Notwithstanding the documented advances, only 38% (25–48%) of the HIV positive children eligible for ART were estimated to receive the treatment in 2016 [4].

In June 2013, Mozambique aligned its PMCT policies to those recommended by WHO [5], implying the implementation of “Option B+” consisting of lifelong ART for all HIV infected and breastfeeding women regardless of their CD4 count and/or clinical status as well as ART being administered to all HIV infected children < 5 years old, independently from CD4 cell count and/or clinical status. In order to guarantee the sustainability of Option B+, Mozambican health authorities implemented a task-shifting (TS) from clinicians to Maternal and Child Health (MCH) nurses within a one stop model (OSM) of care delivered by the same provider in the same consultation. Indeed, until June 2013, in Mozambique, after the identification of the HIV status of the infected child within At Risk Children Clinics (ARCC), the HIV infected mother and her child were referred to an HIV health service (HHS) integrated within the outpatients department (OPD) for a further follow up, where a clinical officer took care of the child and a nurse of the mother.

Beira is the capital of Sofala province, one of the 5 Mozambican provinces in the country with the highest HIV prevalence (15.5%) [6]. In 2013, the coverage of paediatric ART in Sofala was at 32%, so below the national average, and ART initiation for children only reached 70% of the expected provincial target for that same year [6]. In June 2013, in an effort to improve ART initiation and retention of HIV infected children, Beira District Directorate of Health decided to provide the continuum of care for newly HIV positive children < 5 years old and their mothers within the ARCC, postponing their transfer to the HHS integrated by the OPD after the fifth year of age.

The aim of the study was to evaluate a sample of LHFs from the Beira District and determine the impact of the implementation of a different approach to the organization of human health resources based on TS from the clinical officers to nurses in regard to the effectiveness of the new pathway of care on i) the taking charge of HIV infected children, and ii) the administering of paediatric ART therapy.

## Methods

### Study setting, design and population

A retrospective pre-post intervention study was conducted [7].

The LHFs’ inclusion criteria defined by the study protocol included: 1) to have implemented HHS since June 2012; 2) shifting to Option B+ and OSM in June 2013, including ART administered to children within the ARCC.

Of the 15 LHFs of the Beira District, 5 (33.3%) met the previous criteria and were enrolled in the study: Ponta Gêa, Munhava, Macurungo, Nhaconjo, Mascarenha.

All the HIV infected children < 5 years old accessing the ARCC of one of the 5 LHFs between June 2012 to May 2014 were identified through the Antiretroviral (ARV) registries and were recruited in the study. In particular, HIV Exposed Infants (HEI) were identified as HIV infected according to WHO guidelines: through a polymerase chain reaction using a dried blood spots test (PCR-DBS) if less than 18 months old, or through a HIV rapid test if older than 18 months.

Since health data of all children accessing the ARCC of the LHFs who met the study inclusion criteria were available, no sample size calculation was performed.

### Intervention

The health district-level intervention of interest, introduced in Beira by the Local Health Authorities in June 2013, consisted in the TS of the care and treatment of HIV infected children from clinical officers to MCH nurses to ensure a continuum of care within the ARCC.

As the intervention was introduced in June 2013, two time periods lasting 365 days each were compared: one pre-intervention, from June 2012 to May 2013 (T1), and one post-intervention, from June 2013 to May 2014 (T2).

**Fig. 1:**
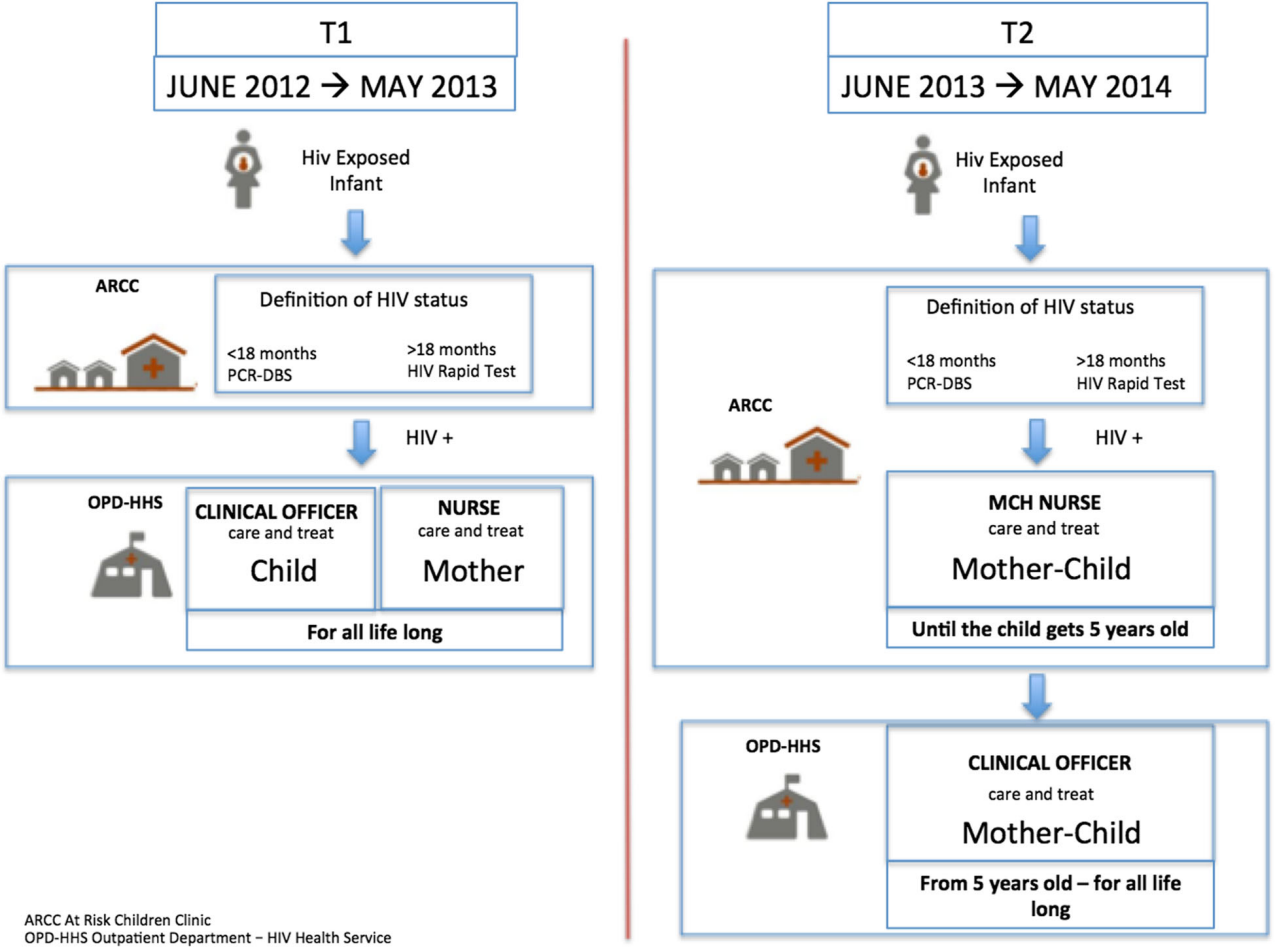
Pathways of care adopted in pre- and post- intervention time periods within the Beira’ HIV healthcare service

Figure 1 shows the pathways of care provided in pre- and post- intervention time periods.

In the pre-intervention pathway of care (pre-TS), all HEIs were followed at the ARCC from the first month of life to the definition of their HIV status. Any child identified as HIV positive was then referred to a HHS integrated within the OPD of the same LHF receiving HIV care and treatment from a clinical officer, occasionally present in the structure, while mothers were under the responsibility of a nurse. In the post-intervention pathway of care (post-TS) all HEIs were monitored at the ARCC until the definitive diagnosis of their HIV status was determined, while any child identified as being infected was hosted in the ARCC until they turned 5 years old. Therefore, HIV infected children and their mothers were monitored and treated by the MCH nurses according to TS. Only after 5 years old could the children, together with the mothers, be referred to the OPD-HHS.

### Endpoints

We compared the post intervention group (post-TS) with the pre intervention group (pre-TS) by using the following set of endpoints as a proxy to assess the impact of the new pathway of care, including TS implementation, on the effectiveness of taking charge of patients:

- ART initiation according to the national guidelines:

1. initiation of ART before/after 9 months; 2. the mean time from the referral to initiation of ART; 3. the mean time from the HIV results to initiation of ART; 4. the mean age at initiation of ART;

b) Cotrimoxazole (CTZ) prophylaxis against HIV-related infection, Isoniazid (INH) prophylaxis against tuberculosis (TB) both were implemented according to the national guidelines;
c) Assessment of nutritional status at each consultation.

Further, we considered CD4 count at the beginning of ART as an indicator of the process of taking charge of patients’ care.

### Data collection and analysis

Individual-level, routinely collected data were extracted from ARCC registries and HHS patients’ files. Patients’ information were further enriched with pharmacy files.

Record linkage was possible through a unique code assigned to the patient. Data extraction was performed between January and March 2016 by trained personnel of “Doctors with Africa” and the research Centre of Infectious Disease (CIDI) of the Catholic University of Mozambique (UCM). Data entry and management was in charge of CIDI research and was performed by using Microsoft Access 2013.

The variables distribution was explored by frequency tables and descriptive statistics such as mean and median. Comparison of the means of the two groups was done by using the t-student test, when data presented a normal distribution, while comparison of medians was performed using the nonparametric Mann Whitney test. The Chi-square test was used to compare the two groups by categorical variables, while Fisher’s exact test was applied when the expected values were below or equal to 5.

Crude Odds Ratios (ORs) were calculated to measure the association between endpoints and intervention (independent variable), being ORs values > 1 predictive of a more effective care.

Data were analyzed by IBM SPSS Software 23 version (IBM Corp., Armonk, NY, USA). All *p*-values were two-sided and p-value < 0.05 was considered statistically significant.

Ethical approval of the protocol was achieved and (as this study used secondary data) informed patient consent was not required.

## Results

Overall, 588 HIV infected children (F = 51.4%) were recruited in the study. Two-hundred fifty eight children (mean age = 15.9 months; range = 14.1–17.7) belonged to the pre-intervention group and 330 (mean age = 16.8 months; range = 15.4–18.3) to the post-intervention group.

Comparisons of the characteristics between the two groups are shown in Table 1. No differences were observed in HIV infected children’s gender, age, weight, height, breastfeeding (breastfeeding or not, duration of breastfeeding) and nutritional status (normal nutrition or level of malnutrition). Also, no significant differences were documented in the two groups with regard to general information concerning HIV infected parents (age of mother/father, mother/father alive at consultation and HIV positive father). A significant statistical difference (*p*-value: 0.007) was highlighted for PMCT implementation to the mother at the first prenatal visit, being that ART was more frequently administered to mothers in the post-intervention group (31.5% versus 23.9%).

**Table 1:** Characteristics of the 588 HIV infected children afferent to in the 5 Beira’s Local Health Facilities and comparison between pre- and post-intervention groups

Characteristics		All N 588	Post-intervention group (postTS) n (%)	Pre-intervention group (preTS) n (%)	*p*-value
Gender	Female	302 (100.0)	163 (49.4%)	139 (53.9%)	0.281
	Male	286 (100.0)	167 (50.6%)	119 (46.1%)	
Mean Age (months)		588 (100.0)	16.8 ± 13.6	15.9 ± 14.6	0.4
Weight at birth	< 2.5 Kg	62 (100.0)	28 (19.7%)	34 (24.8%)	0.306
	> 2.5 Kg	217 (100.0)	114 (80.3%)	103 (75.2%)	
Breastfeeding	Yes	304 (100.0)	153 (77.3%)	151 (84.4%)	0.082
	No	73 (100.0)	45 (22.7%)	28 (15.6%)	
Duration of breastfeeding (months)		100 (100.0)	10.1 ± 4.6	12.4 ± 6.4	0.053
Weight (Kg)		578 (100.0)	8.1 ± 3.1	7.8 ± 3	
Height (cm)		538 (100.0)	69.6 ± 16.2	67.7 ± 16	
Nutritional status (Weight/ Height)					
Normal		309 (100.0)	163 (55.6%)	146 (63.5%)	0.236
Slight malnutrition		83 (100.0)	47 (16%)	36 (15.7%)	
Moderate malnutrition		56 (100.0)	35 (11.9%)	21 (9.1%)	
Severe malnutrition		75 (100.0)	48 (16.5%)	27 (11.7%)	
PMCT implementation for mother at first prenatal visit					
None		106 (100.0)	64 (32.4%)	42 (23.9%)	0.007
HIV Mono prophylaxis		72 (100.0)	36 (18.3%)	36 (20.5%)	
HIV Bi-prophylaxis		91 (100.0)	35 (17.8%)	56 (31.7%)	
HIV ART prophylaxis		104 (100.0)	62 (31.5%)	42 (23.9%)	
Age of mother		512 (100.0)	26 ± 5.7	26.7 ± 6.1	0.226
Mother alive at consultation	Yes	465 (100.0)	263 (93.3%)	202 (92.7%)	0.7494
	No	35 (100.0)	19 (6.7%)	16 (7.3%)	
Age of father		344 (100.0)	32.6 ± 7.4	32.3 ± 9	0.275
Father alive at consultation	Yes	387 (100.0)	222 (90.2%)	165 (91.2%)	0.748
	No	40 (100.0)	24 (9.8%)	16 (8.8%)	
HIV positive father	Yes	101 (100.0)	61 (71.8%)	40 (65.6%)	0.722
	No	45 (100.0)	24 (28.2%)	21 (34.4%)	

Table 2 summarizes the results of the further comparison between the pre- and post-intervention groups by clinical and laboratory information of the 588 HIV infected children obtained by the LHF registries.

**Table 2:** Clinical and laboratory information provided for the 588 HIV infected children afferent to the 5 Beira’s Local Health Facilities: comparison between the pre- and post-intervention groups

Clinical and laboratory information		All N 588	Post-intervention group postTS n (%)	Pre-intervention group preTS n (%)	p-value
ART prophylaxis at birth	Yes	292	146 (80.7%)	146 (70.9%)	0.026
	No	95	35 (19.3%)	60 (29.1%)	
AFB test	Positive	21	7 (4.4%)	14 (7%)	0.31
	Negative	338	151 (95.6%)	187 (93%)	
TB treatment	Yes	51	13 (5.6%)	38 (13.8%)	0.002
	No	457	220 (94.4%)	237 (86.2%)	
Haemoglobin test at initiation of ART	Yes	236	77 (32.6%)	159 (51.8%)	0.027
	No	307	159 (67.4%)	148 (48.2%)	

Significant statistical differences between the two groups were observed with regard to ART prophylaxis at birth (*p*-value: 0.026), being more frequent in the post-intervention group (80.7% versus 70.9%), to tuberculosis (TB) treatment (p-value: 0.002), resulted less frequently in the post-intervention group (5.6% versus 13.8%), and to Haemoglobin test at the initiation of ART (*p*-value: 0.027), less practiced in the post-intervention group (32.6% versus 51.8%). Furthermore, no difference was documented for Acid Bacilli Fast (AFB) test (*p*-value: 0.31) used to detect positivity to TB.

Table 3 reports the comparison between the post and pre intervention groups by the explored endpoints of taking charge of the 588 HIV infected children afferent to the 5 Beira’s LHFs. The initiation of ART after the 9th month of life was documented with a frequency of 54.5% (n.180) in the intervention group and 56.9% (n.147) pre-intervention group, respectively, but no statistical difference was highlighted (*p*-value: 0.703). The mean age in months of HIV infected children at the beginning of ART was 17.2 (± 14.1) for the intervention group and 18.1 (± 15.6) for the pre-intervention group, but no statistical difference was documented (*p*-value: 0.835).

**Table 3:** Endpoints explored in the taking charge of the 588 HIV infected children afferent to the 5 Beira’s local health facilities: comparison between the pre- and post-intervention groups

Endpoints	All	Post-intervention group (postTS)	Pre-intervention group (preTS)	Crude-OR	95% CI	*p*-value
	N 588					
Initiation of ART						
< 9 months	167	95 (28.8)	72 (27.9)	1.08	0.74–1.57	0.703
≥ 9 months	327	180 (54.5)	147 (56.9)			
Missing	94	55 (16.7)	39 (15.2)			
Mean time from referral to initiation of ART						
	494	1.1 (± 5.0)	2.3 (± 4.4)	–	–	0.000
Missing	94					
Mean time from HIV results to initiation of ART						
	359	2.1 (± 4.8)	1.7 (±3.8)	–	–	0.028
Missing	229					
Mean age at initiation of ART						
	494	17.2 (± 14.1)	18.1 (± 15.6)	–	–	0.835
Missing	94					
CTZ prophylaxis						
Yes	528	296 (89.7)	232 (89.9)	1.04	0.54–1.99	0.896
No	40	22 (6.7)	18 (7.0)			
Missing	20	12 (3.6)	8 (3.1)			
INH prophylaxis						
Yes	128	94 (28.5)	34 (13.2)	2.69	1.7–4.15	0.000
No	420	213 (64.5)	207 (80.2)			
Missing	40	23 (7.0)	17 (6.6)			
Regular nutritional visits						
Yes	236	159 (48.2)	77 (29.8)	2.21	1.56–3.15	0.000
No	307	148 (44.9)	159 (61.7)			
Missing	45	23 (6.9)	22 (8.5)			
CD4 count at beginning of ART						
Yes	326	158 (48.0)	168 (65.1)	0.46	0.32–0.65	0.000
No	231	155 (47.0)	76 (29.5)			
Missing	31	17 (5.0)	14 (5.4)			

The mean time from referral to ARCC until the initiation of ART decreased from 2.3 (± 4.4) to 1.1 (± 5.0) months after the implementation of the new pathway of care including TS (*p*-value: 0.000), while the mean time from HIV test results to the initiation of the therapy was lower before the intervention (1.7 ± 3.8 versus 2.1 ± 4.8 months) but no statistical significance was found.

No difference was observed in the CTZ prophylaxis administration, while a significant statistical difference was registered for INH prophylaxis against TB in the post-intervention group, with a documented improvement from 13.2% (n. 34) to 28.5% (n. 94) after the intervention (p-value: 0.000).

After the new pathway implementation, 48.2% (n.159) of HIV infected children underwent regular nutritional assessment as compared to the 29.8% (n.77) of the pre-intervention group (*p*-value: 0.000). On the contrary, the CD4 count at the beginning of ART was performed more frequently in the pre-intervention group with 65.1% (n. 168) versus 48.0% (158) of the post-intervention group (p-value: 0.000).

The most relevant changes highlighted by comparing the pre-post intervention groups (Table 3) resulted in a significant association between the new pathway of care and INH prophylaxis administration against TB (O.R.: 2,69; 95% CI: 1.7–4.15) and regular nutritional assessment during the visits provided in the ARCC (O.R. = 2.21; 95% CI: 1.56–3.15). Again, a significant statistical association was documented between the TS implementation and a decreasing of the CD4 count monitoring at the beginning of ART (O.R. = 0.46; 95% CI: 0.32–0.65).

## Discussion

This pre-post interventional study aimed to retrospectively evaluate the impact on the effectiveness of a new pathway of care for HIV infected children in Mozambique introducing the implementation of TS from clinical officers to MCH nurses.

TS was defined in the First Global Conference as Task Shifting, as a way for the public health communities and national governments to address one of the major constraints to tackling both the HIV/AIDS pandemic and global access to essential health care services [8]. TS refers to transferring tasks to healthcare workers who have not conventionally performed these tasks as part of their practice, (generally, they are more readily available) have completed shorter training and have fewer qualifications [9]. Four levels of TS have been identified based on the extension of the scope of practice of non-physician officers, nurses and midwives, lay health workers or community workers and people living with HIV to self-managed aspects of their care [10].

Particularly, two different pathways of taking charge of HIV infected children and their mothers were compared after the implementation of a second level of TS in 5 LHFs of the Beira district, according to an OSM approach consisting in patients’ care delivered by the same provider in the same consultation in order to guarantee a continuum of care. In fact, the old pathway of care, characterized by the transfer to a different level of health service together with the changing of providers, was assumed to be responsible for a possible delay in taking charge and initiating ART as well as in the retention of care of the newly identified HIV infected children. Such a second level of TS for HIV health care from physicians to nurses has already been demonstrated to be effective, or, no different to the quality of care in Mozambique [11] and in African countries [12–15]. Moreover, a recent review documented no significant difference in the effectiveness of care provided by doctors as compared to nurses on paediatric HIV care [16], but evidence supporting TS of HIV paediatric care is still limited.

Comparing pre and post intervention pathways, we observed some relevant statistical differences. In particular, ART prophylaxis at birth resulted as being practiced at a higher frequency in the post-intervention group, documenting values higher than the expected provincial target for 2013 [6]. Moreover, according to the explored endpoints, an improvement after the TS implementation was highlighted in terms of decreasing in mean time from referral to ARCC until the initiation of ART. Anyway, these results should be re-interpreted in light of the evidence of a more frequent distribution of mothers of HIV infected children of the post-intervention group subjected to ART prophylactic treatment at the first prenatal visit.

Furthermore, increases in regular nutritional assessments during visits after the enrollment in HIV service and in INH prophylaxis rates during the post-intervention period were reported. The effectiveness of the new pathway of care with regard to monitoring malnutrition and the prophylaxis against tuberculosis, respectively, was also documented. On the other hand, the less frequent TB treatment delivered in the post-intervention group suggested a potential controversial impact of the intervention on the global clinical management of concurrent co-morbidities.

It remains controversial how the new pathway of taking charge of HIV infected children resulted in a significant decreasing of CD4 count and Haemoglobin monitoring at the beginning of ART. One explanation of this difference is that performances require more time and commitment as compared to the simple administration of a single therapy and being that the CD4 count was not necessary for the initiation of ART within the Option B+ scenario, it could have been neglected in the post-intervention periods.

Moreover, having been Option B+ and TS implementation concomitant, our study design is not able to isolate the different effects of each specific factor.

For the aforementioned reasons, it is difficult to make a reliable interpretation of the whole result and this represents the main drawback of the study.

Other limitations of our results could be linked to the limited time period available for the comparison - which should be extended in order to extensively assess the effectiveness of the intervention - and to the retrospective nature of the study. In fact, even if pre-post intervention study is often the most feasible option for conducting public health impact evaluations in real world settings, particularly for exploring health services, socio-economic, political or ethic domains, the availability of data is not collected for the specific study, but derives from registries, which is often a major barrier in using it in a retrospective manner [17].

Moreover, records from LHF registries were often incompletely filled in and poorly maintained, mainly because health workers were steadily overloaded, without enough time – and probably without proper training - to provide an adequate completion of health records. As a consequence, for many patients the records were unavailable or incomplete, resulting in missing data for some variables. In addition, the low number of HEI accessing the health services [6] contributed to the restriction of the number of patients enrolled.

Furthermore, the difficulty in controlling important confounding variables, due to the lack of randomization typical of pre-post intervention design [18–20], could not be balanced with an appropriate statistical analysis because of the overall low quality of the database available.

Lastly, no evidence was provided of the availability of a suitable screening protocol for other relevant communicable diseases, such as hepatitis, to implement appropriate health control strategies in the local health settings [21].

However, to our knowledge, this is the first study assessing the effectiveness of TS from clinical officers to nurses in delivering paediatric antiretroviral treatment to Mozambique, and trying to increase the value of data available from local health facilities.

According to available literature, it is clear that TS is a potentially effective approach to address the human resource limitations and to scale up the ART coverage and retention in care for both adults and children [16, 21, 22], however, for a more comprehensive evaluation of quality of care provided, more attention should be paid to human attitude, motivation and training of the personnel involved [23, 24].

## Conclusions

According to our findings, the proposed new pathway of taking charge of HIV infected children under 5 years old supports the provision of the continuum of care. Furthermore, the introduction of the TS from physicians to MCH nurses may have a positive impact on the management of patients affected by HIV and other relevant infectious diseases in the considered context, in particular a decrease in the mean time from the referral to ARCC to the initiation of ART and an increase in Isoniazid (INH) prophylaxis were observed. On the contrary, our findings were controversial for the performance of the nutrition assessment and the CD4 count at the initiation of ART. This suggests how, even if it could be considered appropriate and sustainable to involve nurses in the care of all HIV patients, including both children and their mothers, TS alone may be considered to play a role in the improvement of the global effectiveness of care for HIV infected children only if integrated in a wider range of public health measures including specific pre- and post-lauream education programs for physicians and nurses [25–28].

In conclusion, further studies conducted on a larger scale and in a longer time period are needed to assess the extensive impact of TS on both specific clinical outcomes and on retention in care of HIV infected children in developing countries.

## Abbreviations

AFB: Acid Bacilli Fast
ARCC: At Risk Child Clinic
ART: Antiretroviral treatment
ARV: Antiretroviral
CIDI: Centre of Infectious Disease
CNBS: Comité Nacional de Bioética para Saúde
CTZ: Cotrimoxazole
HEI: HIV Exposed infants
HHS: HIV Health Service
HIV: Human Immunodeficiency Virus
INH: Isoniazid
LHF: Local health facilities
MCH: Mother and Child Health
OPD: Outpatients department
OSM: One stop model
PCR: DBS Polymerase chain reaction using dried blood spots
PMCT: Prevention of Mother to Child Transmission
TB: Tuberculosis
TS: Task Shifting
UCM: Catholic University of Mozambique
WHO: World Health Organization
